# Supplementary material for: Occupational health risk of farmers: A qualitative study with the agriculture society of trinidad and tobago and the ministry of agriculture, land and fisheries
Source: PLoS One. 2025 Jul 2;20(7):e0327283. doi: 10.1371/journal.pone.0327283 (PMC12221002; doi:10.1371/journal.pone.0327283)
Supplement: Attachment — Submitted filename: PONE-D-24-46271_reviewer.pdf [file pone.0327283.s001.pdf]

PLOS ONE

Occupational Health Risk of Farmers: A Qualitative Study With the Agriculture Society of Trinidad and Tobago and the Ministry of Agriculture, Land and Fisheries.

--Manuscript Draft--

**Manuscript Number:**	PONE-D-24-46271
**Article Type:**	Research Article
**Full Title:**	Occupational Health Risk of Farmers: A Qualitative Study With the Agriculture Society of Trinidad and Tobago and the Ministry of Agriculture, Land and Fisheries.
**Short Title:**	Occupational Health Risk of Farmers
**Corresponding Author:**	Avishek Choudhury, Ph.D.<br>West Virginia University<br>Morgantown, WV UNITED STATES OF AMERICA
**Keywords:**	Safer choice products; occupational safety; toxic chemical; food safety; health risk
**Abstract:**	The agriculture sector is regarded as one of the most hazardous sectors and it is significantly affected by pesticide toxicity leading to neurological diseases. respiratory illnesses and cancers. The objective of this research is to explore the perception of the representatives of the Agriculture Society of Trinidad and Tobago (ASTT) and the officials of the Ministry of Agriculture, Land and Fisheries (MALF) on the protection of farmers particularly on the exposure to pesticides in Trinidad and Tobago. We conducted 30 minute long semi structured interviews with 32 individuals from the MALF and ASTT. Deidentified interview transcripts were analyzed using a combination of inductive and deductive thematic analysis. Responses reveal 6 critical themes related to farming practices, chemical usage, and safety measures: (a) Barriers to Proper Use of Personal Protective Equipment (PPE); (b) Challenges in Safe Chemical Use and Disposal Practices; (c) Health Impacts of Unsafe Chemical Exposure, (d) Challenges in Farmer Training and Adoption of Safety Practices; (e) Lack of Policies and Monitoring for Farmer Safety and Chemical Use; (f) Absence of Quality Control and Unsafe Practices in Local Produce. Our research highlights the urgent need for better safety regulations, more effective training, and the establishment of monitoring systems to protect the health of farmers and ensure safe agricultural practices. By shedding light on these issues, this study paves the way for the development of targeted interventions that can improve farmer health and safety standards in Trinidad and Tobago's agricultural sector.
**Order of Authors:**	Barry Parasram
	Avishek Choudhury, Ph.D.
**Additional Information:**	
**Question**	**Response**
**Financial Disclosure**<br><br>Enter a financial disclosure statement that describes the sources of funding for the work included in this submission. Review the submission guidelines for detailed requirements. View published research articles from *PLOS ONE* for specific examples.<br><br>This statement is required for submission and **will appear in the published article** if the submission is accepted. Please make	The author(s) received no specific funding for this work.

Powered by Editorial Manager® and ProduXion Manager® from Aries Systems Corporation

sure it is accurate.<br><br>**Funded studies**<br>Enter a statement with the following details:<br>• Initials of the authors who received each award<br>• Grant numbers awarded to each author<br>• The full name of each funder<br>• URL of each funder website<br>• Did the sponsors or funders play any role in the study design, data collection and analysis, decision to publish, or preparation of the manuscript?<br><br>Did you receive funding for this work?	
**Competing Interests**<br><br>Use the instructions below to enter a competing interest statement for this submission. On behalf of all authors, disclose any competing interests that could be perceived to bias this work—acknowledging all financial support and any other relevant financial or non-financial competing interests.<br><br>This statement is required for submission and **will appear in the published article** if the submission is accepted. Please make sure it is accurate and that any funding sources listed in your Funding Information later in the submission form are also declared in your Financial Disclosure statement.<br><br>View published research articles from *PLOS ONE* for specific examples.	The authors have declared that no competing interests exist.

**NO authors have competing interests**<br><br>Enter: *The authors have declared that no competing interests exist.*<br><br>**Authors with competing interests**<br><br>Enter competing interest details beginning with this statement:<br><br>*I have read the journal's policy and the authors of this manuscript have the following competing interests: [insert competing interests here]*<br><br>* typeset	
**Ethics Statement**<br><br>Enter an ethics statement for this submission. This statement is required if the study involved:<br><br>• Human participants<br>• Human specimens or tissue<br>• Vertebrate animals or cephalopods<br>• Vertebrate embryos or tissues<br>• Field research<br><br>Write "N/A" if the submission does not require an ethics statement.<br><br>General guidance is provided below. Consult the submission guidelines for detailed instructions. **Make sure that all information entered here is included in the Methods section of the manuscript.**	The study received approval from the Institutional Review Board of West Virginia University (Protocol #2401909110).<br><br>Informed consent was obtained from all individual participants included in the study via cover letter.

**Format for specific study types**<br><br>**Human Subject Research (involving human participants and/or tissue)**<br>• Give the name of the institutional review board or ethics committee that approved the study<br>• Include the approval number and/or a statement indicating approval of this research<br>• Indicate the form of consent obtained (written/oral) or the reason that consent was not obtained (e.g. the data were analyzed anonymously)<br><br>**Animal Research (involving vertebrate animals, embryos or tissues)**<br>• Provide the name of the Institutional Animal Care and Use Committee (IACUC) or other relevant ethics board that reviewed the study protocol, and indicate whether they approved this research or granted a formal waiver of ethical approval<br>• Include an approval number if one was obtained<br>• If the study involved *non-human primates*, add *additional details* about animal welfare and steps taken to ameliorate suffering<br>• If anesthesia, euthanasia, or any kind of animal sacrifice is part of the study, include briefly which substances and/or methods were applied<br><br>**Field Research**<br><br>Include the following details if this study involves the collection of plant, animal, or other materials from a natural setting:<br>• Field permit number<br>• Name of the institution or relevant body that granted permission	
**Data Availability**<br><br>Authors are required to make all data underlying the findings described fully available, without restriction, and from the time of publication. PLOS allows rare exceptions to address legal and ethical concerns. See the PLOS Data Policy and FAQ for detailed information.	Yes - all data are fully available without restriction

A Data Availability Statement describing where the data can be found is required at submission. Your answers to this question constitute the Data Availability Statement and **will be published in the article**, if accepted.<br><br>**Important:** Stating 'data available on request from the author' is not sufficient. If your data are only available upon request, select 'No' for the first question and explain your exceptional situation in the text box.<br><br>Do the authors confirm that all data underlying the findings described in their manuscript are fully available without restriction?	
**Describe where the data may be found in full sentences. If you are copying our sample text, replace any instances of XXX with the appropriate details.**<br><br>• If the data are **held or will be held in a public repository**, include URLs, accession numbers or DOIs. If this information will only be available after acceptance, indicate this by ticking the box below. For example: *All XXX files are available from the XXX database (accession number(s) XXX, XXX.).*<br>• If the data are all contained **within the manuscript and/or Supporting Information files**, enter the following: *All relevant data are within the manuscript and its Supporting Information files.*<br>• If neither of these applies but you are able to provide **details of access elsewhere**, with or without limitations, please do so. For example:<br><br>*Data cannot be shared publicly because of [XXX]. Data are available from the XXX Institutional Data Access / Ethics Committee (contact via XXX) for researchers who meet the criteria for access to confidential data.*<br><br>*The data underlying the results presented in the study are available from (include the name of the third party*	All XXX files are available from the XXX database (accession number(s) XXX, XXX.).

and contact information or URL).<br>• This text is appropriate if the data are owned by a third party and authors do not have permission to share the data.<br><br>* typeset	
Additional data availability information:	Tick here if the URLs/accession numbers/DOIs will be available only after acceptance of the manuscript for publication so that we can ensure their inclusion before publication.

Powered by Editorial Manager® and ProduXion Manager® from Aries Systems Corporation

Click here to access/download;Manuscript;Manuscript.docx

Occupational Health Risk of Farmers: A Qualitative Study With the Agriculture Society of Trinidad and Tobago and the Ministry of Agriculture, Land and Fisheries.

Barry Parasram [1,2,a] and Avishek Choudhury [1,3,b]

[1]Occupational Safety and Health, West Virginia University, USA
[2] Cipriani College of Labour and Cooperative Studies, Valsayn, Trinidad & Tobago.
[3]Industrial and Management Systems Engineering, West Virginia University, USA

[a] bp00052@mix.wvu.edu
[b] avishek.choudhury@mail.wvu.edu; https://orcid.org/0000-0002-5342-0709

Corresponding:
Avishek Choudhury (Ph.D.)
Assistant Professor
Industrial and Management Systems Engineering
Benjamin M. Statler College of Engineering and Mineral Resources
West Virginia University
1306 Evansdale Drive
Morgantown, West Virginia 26506
USA
avishek.choudhury@mail.wvu.edu;

Statements & Declarations

Funding
The authors declare that no funds, grants, or other support were received during the preparation of this manuscript.

Competing Interests
The authors have no relevant financial or non-financial interests to disclose.

Author Contributions
All authors contributed to the study conception and design. Material preparation, data collection and analysis were performed by Barry Parasram and Avishek Choudhury. The first draft of the manuscript was written by Barry Parasram and all authors commented on previous versions of the manuscript. All authors read and approved the final manuscript.

Ethics approval
The study received approval from the Institutional Review Board of West Virginia University (Protocol #2401909110).

Consent to participate
Informed consent was obtained from all individual participants included in the study.

Occupational Health Risk of Farmers: A Qualitative Study With the Agriculture Society of Trinidad and Tobago and the Ministry of Agriculture, Land and Fisheries

Abstract

The agriculture sector is regarded as one of the most hazardous sectors and it is significantly affected by pesticide toxicity leading to neurological diseases. respiratory illnesses and cancers. The objective of this research is to explore the perception of the representatives of the Agriculture Society of Trinidad and Tobago (ASTT) and the officials of the Ministry of Agriculture, Land and Fisheries (MALF) on the protection of farmers particularly on the exposure to pesticides in Trinidad and Tobago. We conducted 30 minute long semi structured interviews with 32 individuals from the MALF and ASTT. Deidentified interview transcripts were analyzed using a combination of inductive and deductive thematic analysis. Responses reveal 6 critical themes related to farming practices, chemical usage, and safety measures: (a) Barriers to Proper Use of Personal Protective Equipment (PPE); (b) Challenges in Safe Chemical Use and Disposal Practices; (c) Health Impacts of Unsafe Chemical Exposure, (d) Challenges in Farmer Training and Adoption of Safety Practices; (e) Lack of Policies and Monitoring for Farmer Safety and Chemical Use; (f) Absence of Quality Control and Unsafe Practices in Local Produce. Our research highlights the urgent need for better safety regulations, more effective training, and the establishment of monitoring systems to protect the health of farmers and ensure safe agricultural practices. By shedding light on these issues, this study paves the way for the development of targeted interventions that can improve farmer health and safety standards in Trinidad and Tobago's agricultural sector.

Keywords: *Safer choice products; occupational safety; toxic chemical; food safety; health risk*

INTRODUCTION

Pesticides are environmental contaminants that are specifically designed to control pests and diseases. However, these chemicals which are intended to uplift agricultural practices have been responsible for an estimated 200,000 acute poisoning deaths every year where 99% occur in low and middle-income countries[1], primarily affecting farmers. The World Health Organization (WHO) estimates that there is a further one million unintentional pesticide poisoning occur annually leading to 20,000 deaths[2]. The agriculture industry which accounts for over a billion workers [3] is significantly affected by pesticide toxicity [4]. There are significant concerns over the toxic effects of pesticides on humans where linkages to neurological disease have been noted [5] making farmers susceptible to ailments including lymphocytic leukaemia, non-Hodgkin's lymphoma, lung cancer, and other melanoma skin cancer [67]. Studies have outlined exposure to pesticides as an increased risk of Parkinson's disease [8] and cognitive impairment [9].

Despite the known risks of pesticide use among farmers, its negative impact is particularly heightened in underserved regions. Low and middle-income countries (LMICs) often lack safety policies and their implementation due to limited resources [1010]. It has been seen that many LMICs allow inappropriate use of toxic pesticides that are banned in the European Union (EU) and the United States of America (US) [11].

It is important to understand that farmers are a vulnerable group that contributes to an integral part of the welfare worldwide. While there is greater knowledge of the hazards in the sector, they are not protected. As a result, the protection of the workers should be one of the priorities across LMICs. These workers have a general lack of awareness about the safe use of pesticides and its health hazards. Therefore, in-depth research in occupational safety and health among farmers are needed.

In this study we focus on the health and occupational hazards of farmers in Trinidad and Tobago. The exposure to pesticides in this nation is significant where the farmers experience acute occupational pesticide poisoning resulting from little to no training on safe pesticides and their usage [12]. In a recent survey of 208 farmers, about 47 % of the participants stated that they felt unwell within 24 hours of pesticide use [13]. Workers in the sector are involved in practices where no preventive measures are undertaken and an acceptance that adverse health effects of agricultural work are expected [14]. The health and safety issues are exacerbated by the lack of policies and lack of enforcement of regulations related to pesticides. Furthermore, in Trinidad and Tobago, data on agricultural injuries and accidents is unavailable, magnifying the pesticide exposure predicament. There is also a lack of studies exploring the long-term effects of toxic pesticide use on farmer and the consumers of produce.

The objective of this research is to explore the perception of the representatives of the Agriculture Society of Trinidad and Tobago (ASTT) and the officials of the Ministry of Agriculture, Land and Fisheries (MALF) on the protection of farmers particularly on the exposure to pesticides. The findings of this research will help contribute to policy-making by providing evidence from research on the state of the sector.

METHOD

Ethics statement

The study received approval from the Institutional Review Board of West Virginia University (Protocol #2401909110). No identifiers were collected during this study.

Study site

According to the World Bank, Trinidad and Tobago has a population of 1534937 with a population growth of 0.254 and a life expectancy of 74. Its 10.5% of the land area is designated as agricultural land with a contribution of 1.07% towards GDP [15]. In Trinidad and Tobago, the Ministry of Agriculture, Land and Fisheries (MALF) champions biodiversity conservation and

sustainable development of food and non-food systems. It is responsible for delivering integrated services aligned with a changing food and agriculture system [16]. Within the MALF exists an agency founded in 1839 named the Agriculture Society of Trinidad and Tobago (ASTT) that represents all farmers. It is a statutory body within the MALF representing all sectors, accounting for the 32774 farmers [17]. A total of 28 442 farmers are located across 8 counties in Trinidad and 4332 in the sister island of Tobago. Each county has multiple districts where an extension officer from the MALF is assigned to specific districts in the region. An extension officer provides technical information on various areas from the farmer registration process and agricultural technologies. Tobago is managed by the Division of Agriculture under the Tobago House of Assembly.

Study Design

The study is underscored using phenomenology. This approach delved into the experience of the participants capturing their insights and attitudes towards pesticide use as well as the impact on their farming practices and health. Based on the problem identified and the area of research, a qualitative method approach was best suited. Interviews were conducted until data saturation. Individuals from ASTT and MALF were invited to voluntarily participate in the study. A purposive sampling was used as participants were chosen due to their experience and knowledge in the sector-specific to the objective of the study. The representatives of the MALF and ASTT were interviewed with approval from the Permanent Secretary. A request letter was sent to the Permanent Secretary on February 21^{st,} 2024, and March 4^{th,} 2024, respectively.

All interviews were done face to face in person at the various offices of MALF during the period March 25th- April 12^{th,} 2024. Participants from ASTT were interviewed during the period March 5th – March 20th in person and online setting. The interviews lasted approximately 30 minutes each. In addition, one interview was done with a representative of a local well-

established chemical manufacturer. This company was contacted on March 24th and the interview was conducted on March 26th, face-to-face in person.

Data Collection

An interview guide (Table 1) was created to assist in the collection of data in the interview process. All interviews were done privately with no interference.

Table 1. Interview guide.

Leading Questions	Probing Questions
What is your overall assessment of the Agriculture Sector both as a viable sector and its importance to the economy of the country?	Describe the responsibility of your organisation as it pertains to the Agriculture Sector.
What are the Occupational Safety and Health challenges in the Agriculture Sector?	Are you aware of the ways to deal with these challenges?
What assistance, guidance and training do you provide to farmers ?	How often are these training done?<br>How do you determine what training interventions are needed?
What are the policies, systems or measures are in place to protect farmers against Exposure to pesticides, Pests, and Diseases	Are farmers aware of measures to deal with the issues of pesticide exposure, harmful chemicals and pests and diseases? Are farmers aware of proper ways to store and dispose of pesticides and other harmful chemicals?
Describe the specific safety regulations and guidelines in place for farmers?	Are there written standards in place to guide farmers?
How are safety standards monitored and enforced in agricultural operations?	What unique safety challenges are usually reported by farmers? How do you address these challenges?
What programs or initiatives exist to educate farmers about safety practices and procedures?	Are you aware when these education and training workshops are held? How is it held? Is it in person or online? Do you have access to an online platform for the online training?

Interviewer

The field interviews were led by the author, resident of the study site and is a senior lecturer of Occupational Health and Safety (OSH). The interviewer is a male and holds qualifications namely MSc in OSH and CertIOSH. He is a trained interviewer and is familiar with the local culture, language, and safety issues. Before the commencement of the interviews, the participants were informed of the professional background of the researcher and the reasons

for conducting the research. They were also notified of the importance of gathering information in the subject area due to the need to influence a healthier worker in a safe environment. A prepared letter was shared with the participants giving an overview of the study and the protocol number stating the IRB approval. Sufficient time was also spent in the field gaining more experience with the participants in their settings. The researcher developed an in-depth understanding, and this enabled the conveyance of details about the site and people thus lending credibility. The interviews were conducted using a prepared interview protocol and recorded for further use in the data analysis process.

Data Analysis

All interviews were audio recorded with permission from the participants. The recordings were uploaded to a secure cloud server and each was transcribed using the software 'Whisper'[18]. It is an automatic speech recognition system that enables transcription in multiple languages. The transcriptions were also manually corrected for spelling and other errors. Then, identifiers such as names of persons and places were removed from the text.

The data were analyzed using a combination of inductive and deductive thematic analysis[19] to ensure a comprehensive understanding of the key themes and patterns in the study. Initially, two authors independently reviewed the transcripts, engaging in a thorough examination to gain an in-depth understanding of the content and the context surrounding the interviews. This process allowed for the emergence of both anticipated and unanticipated themes, providing a robust framework for further analysis. The primary focus of the analysis was on pesticide usage and safety among farmers, exploring how different stakeholders, including farmers themselves, members of the Ministry of Agriculture, and pesticide manufacturers, perceive and address these issues. The inductive phase allowed the authors to identify recurring themes directly from the data without preconceived notions, while the

deductive approach facilitated the validation and organization of these themes based on existing literature and frameworks related to pesticide use and safety.

During the analysis, specific attention was given to the different perspectives of the stakeholders, such as the challenges farmers face with pesticide application, the regulatory and advisory roles of the Ministry of Agriculture, and the role of pesticide manufacturers in ensuring product safety and efficacy (if any).

RESULTS

Participants

A total of 32 interviews were conducted with participants both representing the ASTT and the MALF. A total of 17 participants were MALF Officers, 14 were farmers (ASTT), and 1 Technical Officer in a chemical company. Table 2 shows the participant characteristics.

Table 2. Participant characteristics (n=32)

	**N(%)**
**Sex**	
Female	19 (59)
Male	13 (41)
**Age Category**	
18-35	6 (19)
36-55	21 (66)
56-65	2 (6)
66 and over	3 (9)
**Groups**	
Ministry of Agriculture (MA)	17 (53)
Farmer Group (FG)	14 (44)
Chemical Company (CC)	1 (3)

Themes

Responses from participants reveal several critical themes related to farming practices, chemical usage, and safety measures. Key themes include Barriers to Proper Use of Personal Protective Equipment (PPE), where participants highlight the challenges in adopting safety gear due to tradition, lack of knowledge, and urgency. The theme of Challenges in Safe

Chemical Use and Disposal Practices emerges as participants discuss the misuse of chemicals and improper disposal methods that pose environmental risks. Another theme, Health Impacts of Unsafe Chemical Exposure, underscores the serious health consequences, such as cancer and skin diseases, linked to unsafe chemical handling. In Challenges in Farmer Training and Adoption of Safety Practices, participants point out issues with the timing, content, and engagement in training programs, particularly concerning health and safety. Lack of Policies and Monitoring for Farmer Safety and Chemical Use addresses the absence of regulatory frameworks and enforcement mechanisms to ensure safe practices. Finally, the theme of Absence of Quality Control and Unsafe Practices in Local Produce reveals concerns about the lack of monitoring for chemical residues in locally sold crops, with some participants favoring imported produce over local due to safety concerns. Together, these themes underscore the need for improved safety practices, training, policy development, and quality control in the agricultural sector.

Barriers to Proper Use of Personal Protective Equipment (PPE) Among Farmers.

This theme highlights various challenges that farmers face in adopting the proper use of PPE, such as lack of awareness, tradition, urgency, and mindset, as discussed by participants from different sectors, including farmers, pesticide manufacturers, and the Ministry of Agriculture. The participants point out issues like inadequate precautionary measures, resistance to change from traditional practices, lack of education and knowledge about PPE, and farmers' perceived comfort in not using protective gear.

- Participant 4 FG: *'There's nothing, you may see a farmer sometimes wear a jersey over his face and that's just because of the wind blow, you know, but there's not a lot of precautionary measures'*
- Participant 6 FG: *'You still have farmers who will go there and not use the mask. You still have farmers who would not use a chemical mask or a dust mask. You still have farmers*

who would not use gloves. You still have farmers who would walk in the field bare feet. Most of them wouldn't wear a mask. They may wear a head cover. Some of them have a bandana or something like that. But they wear boots and not long pants. Some of them wear short sleeves. So they're still exposed'

- Participant 14 CC*: 'We go around in the field although we sell pesticides, we still stress the use of proper PPE, respirators, gloves, and boots but based on tradition it's very difficult to change a person's mindset from doing things for years, generational farming for years, doing wrong practices, actually spraying with the wind or against the wind without using respirators, masks, and even gloves to mix or handle the pesticides'*
- Participant 32 MA*: 'I think the number one, the number one problem I notice or feel is the incorrect use of pesticides and chemicals. The lack of problems farmers utilize, which is not using protective gear, proper PPE, more for better problems, most of them may not, they simply don't have the information, they lack the education, they lack the knowledge, they lack the advantages of using protective gear and some of them are just holding on to traditional based methods, which is, you know, simply put spraying chemicals without even respirators, without gloves, without boots and they do not see the importance of health and safety when they're on the field'*

Challenges in Safe Chemical Use and Disposal Practices.

This theme emphasizes the difficulties related to the misuse of chemicals, improper handling, and the environmental impacts of unsafe disposal of chemical containers. Several participants highlight issues like improper chemical handling, leaving empty chemical bottles in inappropriate places, and the lack of safe disposal practices.

- Participant 12 FG: '*So after using the chemical, they don't dispose of it properly. I, at least know you're supposed to rinse the bottle two to three times. And then you pierce the holes*

and this kind of thing. But from time to time, because as I told you, we have like about 20 farmers. When I go to visit and, help out some of the farmers, I often see these pesticides and weedicide bottles all over. It's just advertising'

- Participant 14 FG: '*Storage is one aspect, but how they store it is a different scenario because they have everything close to each other. And disposal, even disposal as well. For example, when you go down to most of these agriculture farms in large acreages, when they finish with that pesticide bottle, they throw it in the river, which leads to pollution and flooding. There's no organization or no company right now that I'm aware of that takes back empty pesticide plastic bottles to be recycled'*
- Participant 16 MA: *'But in terms of safety aspects, you hardly see any type of change in terms of how they dress. Right. How they dispose and how they store the chemicals. They still store in open buckets or it's still open in the van tray. They still have bare feet. They still have no gloves'*
- Participant 17 MA: *'When you go, you always see, empty bottles and chemical bottles, and I always encourage them, listen, try to dispose of these chemical bottles, don't leave them there, you know, because of the residual effect'.*
- Participant 27 MA*: 'Yes, you go out and you tell them, listen, these are your chemicals. Why are your bottles in the drain? Why is it in the river?'*

However, some participants also demonstrate awareness of safer practices, such as using neutralization methods and minimizing chemical use due to crop type, indicating varying levels of knowledge and practice among farmers.

- Participant 2 FG*: 'I'm aware that if I want to wash a can, you have a charcoal pit where you wash a can and through that access, that wastewater is washed away in the charcoal pit to neutralize the chemical'*
- Participant 6 FG*: 'The crops that they plant, you don't want to use many chemicals. Cocoa*

is not something that you use many chemical'

Health Impacts of Unsafe Chemical Exposure

This theme captures the participants' observations and personal experiences regarding the serious health consequences associated with unsafe pesticides and chemical use in farming. The comments highlight issues such as cancer, heart problems, skin diseases, and even death, which are attributed to long-term exposure to chemicals without proper protective measures. The participants also reflect on the lack of medical attention or seriousness given to these health issues, reinforcing the need for greater awareness and precautionary practices in agriculture.

- Participant 1 FG: *'Yes, more or less the larger farmers who have been involved in agriculture since, let me say about 40 years ago, are now faced with cancer. You would see farmers from time to time having heart issues that are occurring right now'*
- Participant 2 FG: *'I know of a friend who would have passed late last year, a young guy. I always keep telling him the way I see him, the way he exposes himself to chemicals, no gloves, no this, no that. And he died just recently, so to say'*
- Participant 5 FG: *'My mom died 12 years ago and I believe what brought it on, it didn't come on all of a sudden because they were misusing pesticides, they weren't using it safely'*
- Participant 12 FG: *'A lot of farmers have been getting skin disease and this kind of thing, and they're not taking it seriously or they're not even going to the doctor to verify if it is a result of the fertilizers being applied to their crops'*

Challenges in Farmer Training and Adoption of Safety Practices

This theme highlights the difficulties in effectively delivering training programs to farmers, particularly concerning safety and health practices. The responses reveal several barriers, such as poor attendance due to inconvenient training times, reluctance to participate in safety-focused courses, and a lack of training specifically addressing health and safety. While the Ministry of Agriculture, Land, and Fisheries (MALF) and the Agricultural Society of Trinidad

and Tobago (ASTT) provide training and advisory services, there is consensus that more focus is needed on farmer health and safety.

- Participant 3 FG*: 'They might have one training and they will invite the farmers. Many of the farmers do not know'*
- Participant 8 FG*: 'From time to time they will offer training and then grow various crops. But to be honest, I have not seen anything catered specifically to target health and safety and the environment towards the farmers'*
- Participant 9 FG: *'It is very challenging to be in a class from 9 o'clock to 11 o'clock and that's prime work time for a farmer, you know so having these classes doesn't make much of a difference'*
- Participant 21 MA*: most farmers now do have a regular job, so sometimes getting them out for training is a problem*
- Participant 30 MA: *'Not really, we do not do any training on safety and health'.*

Some participants also point out that while training for crop production is emphasized, farmers often avoid or resist safety-related training, viewing it as tedious or unnecessary, further complicating the adoption of safe practices.

- Participant 1 FG; *'They do have the training, but farmers don't adhere to it'.*
- Participant 12 FG*: in terms of safety, full PPE, if you tell a farmer, listen, you need to come to our course so that we can teach you exactly what the full PPE that you should wear when applying these fertilizers the first thing they say is no, no, they don't want to hear that.*
- Participant 25 MA*: 'We do a lot of training in pesticide safety but some of them are hesitant because they think doing the correct thing is tedious for them'*

Lack of Policies and Monitoring for Farmer Safety and Chemical Use

This theme emphasizes the absence of formal policies and monitoring systems that regulate the safe use, handling, and storage of chemicals in the agricultural sector. The responses indicate

that while extension officers provide information, farmers bear full responsibility for safety practices without regulatory oversight. The lack of policies or safety officers to enforce compliance compounds health risks for farmers. Participants expressed a desire for robust policies and better enforcement mechanisms to ensure safe agricultural practices and protect farmer health. The comments also reveal that current guidance is limited to product labels, which is insufficient for ensuring proper chemical usage.

- Participant 5 FG: *I would love to see that policy come about to help the safety of the farmers*
- Participant 8 FG: *I don't think there is any safe use or safe practices for the agriculture sector in effect right now*
- *Participant 15 FG: Based on research I've seen that farmers are suffering long-term from exposure to pesticides particularly because of lack of training lack of education poor farming practices from no monitoring and a lack of a policy*
- Participant 16 MA: *'No, there is no policy or guideline in place for the safe use of chemicals, It's basically when you buy a product, there is a label and it's what the label tells you'*
- Participant 21 MA*: 'There's no policy in place with pesticide usage or anything like that'*
- Participant 26 MA*: 'We don't have any responsibility and we cannot penalize the farmers for not following the guidelines'*

Absence of Quality Control and Unsafe Practices in Local Produce

This theme highlights the lack of monitoring and quality control measures for chemical residues in locally grown produce, raising concerns about food safety for domestic consumers including farmers. While export products are subject to external quality regulations, local produce does not undergo the same scrutiny, leading to unsafe practices such as ignoring pre-harvest intervals and using inappropriate chemicals. Some participants, including farmers, express a preference for imported produce due to concerns about the safety of local crops. The

responses emphasize the need for improved quality assurance and stricter regulations to protect consumer health and maintain confidence in local agricultural products.

- Participant 7 FG: *I just tell my parents or my friends and siblings, when they buy leafy vegetables, especially cabbage, don't buy from Trinidad, buy the imported one"*
- *Participant 12 FG: I think that more has to be done to ensure that when farmers use whatever chemical they do following the pre-harvest interval period. I don't know how they will do it, but they need to have something testing crops, at least randomly testing the crops in the market. And so at least instill some fear in the farmer to know and listen. If your crop tests positive for some chemical that is not supposed to be utilized, there must be some form of penalty.*
- Participant 14 CC: *A lot of the farmers actually would not even observe the pre-harvest interval times of pesticides anyhow so they will use products that have long harvest intervals and harvest the next two or three days which generally for the consumers is very toxic and very harmful to them*
- Participant 16 MA: *'Some farmers want to spray the black disinfectant. Farmers do their own thing. You're telling them not to use products that are not designed for crops, especially on stuff that you use fresh. But they are persistent'*
- Participant 27 MA*: 'I would have gone and the farmer had his cabbage with some other worm bites. So he's fully well aware that the amount of chemicals he's using is dangerous to himself and even others. So he chose to eat it and sell it to the grocery stores as well'*

DISCUSSION

This study makes a significant contribution as the first to explore the impact of pesticide use on farmer health and safety in Trinidad and Tobago, gathering perspectives from both farmers and the Ministry of Agriculture. Our research highlights the urgent need for better safety regulations, more effective training, and the establishment of monitoring systems to protect the

health of farmers and ensure safe agricultural practices. By shedding light on these issues, this study paves the way for the development of targeted interventions that can improve farmer health and safety standards in Trinidad and Tobago's agricultural sector.

PPE Use

Our findings regarding the use of PPE among farmers reveal a significant gap between the availability of safety gear and its actual usage. Despite efforts from extension officers to educate farmers about the importance of PPE, many continue to neglect its use due to ingrained habits, convenience, and a lack of knowledge. This situation is consistent with the literature, which highlights various factors influencing PPE compliance among agricultural workers. Widianto et al. identify several internal factors that affect farmers' ability to recognize hazardous materials, including their age, farming experience, and education level[20]. Similarly, Imoro et al. report that many farmers do not utilize safety gloves or masks during pesticide application, leading to significant health risks from dermal and inhalation exposure[21]. Moreover, the literature indicates that even when farmers are aware of the necessity of PPE, compliance remains low. For instance, Sai et al. found that while many farmers recognized the need for protective gear, actual usage was inconsistent[22]. This finding resonates with our observation that despite the availability of PPE, its use is often compromised by convenience and habitual practices. Furthermore, Odisho and Mohammad highlight that improper PPE usage, alongside unsafe practices like eating or drinking on the farm, significantly increases the risk of pesticide exposure[23]. This suggests that mere availability of PPE is insufficient; there must be a concerted effort to change farmers' mindsets and behaviors towards its use. The literature also emphasizes the role of education in improving PPE compliance. For example, Rosanti et al. advocate for comprehensive education on pesticide safety management, which includes proper PPE usage[24]. This is echoed by Damalas and Koutroubas, who assert that training on pesticide use is associated with elevated safety behaviors among farmers[25]. Our study support this notion, indicating that more targeted educational interventions are necessary

to foster safety culture among farmers. However, some research indicates a positive correlation between education and PPE usage, others report no significant relationship[26,27] .

Pesticide Use and Safe Handling

Our findings regarding pesticide use and disposal reveal a significant issue of improper handling and disposal practices among farmers, which poses considerable environmental and health risks. Participants in the study indicated a tendency to misuse pesticides, particularly by neglecting pre-harvest intervals, and to dispose of chemical containers unsafely. This aligns with existing literature that highlights similar patterns of behavior among farmers in various regions. Research by Sai et al. emphasizes that improper pesticide disposal contaminates soil and water sources, adversely affecting non-target organisms and the broader ecosystem[22]. Similarly, Mergia et al. corroborate these findings, noting that the disposal of leftover pesticides and empty containers is a critical concern, particularly in LMICs[28]. This is echoed by Hashemi et al., who point out that inadequate training and awareness among farmers contribute to unsafe pesticide use and disposal practices, leading to significant health risks. Hashemi et al. also found that farmers with higher education levels exhibited better knowledge and safer behaviors regarding pesticide use[29]. This suggests that targeted educational programs could mitigate the risks associated with improper pesticide handling, as highlighted by multiple studies, including those by Negatu et al. and Mohamed et al[30.31]. The disposal methods reported by participants in our study—such as burning or burying empty containers—are consistent with findings from other regions. For example, Jallow et al., noted that many farmers in Kuwait also adopted hazardous disposal methods, leading to environmental contamination[32]. Such practices not only threaten environmental integrity but also pose direct health risks to farmers and consumers alike, as improper disposal can lead to pesticide residues entering the food chain .

Health Risks

Our findings regarding the health impacts of unsafe chemical exposure among farmers reveal a concerning trend of serious health conditions, including cancer, heart disease, and skin

disorders, attributed to inadequate safety measures and unsafe practices. This aligns closely with the existing literature, which consistently highlights the adverse health effects associated with pesticide exposure in agricultural settings. In a study Kapeleka et al. found that farmers exposed to pesticide mixtures exhibited significant health risks, including compromised liver and kidney functions[33]. Similarly, Karunamoorthi et al. report that a large number of farmers are exposed to harmful pesticides, which are often banned in industrialized countries, further compounding health risks[34]. In addition, the literature suggests that the cumulative effects of long-term pesticide exposure can lead to chronic health conditions. For example, Fibriansari et al. note that farmers with prolonged exposure to pesticides often do not use PPE, resulting in a decline in their immune function and increased susceptibility to various diseases[35]. This aligns with our findings, where participants reported serious health conditions that may be linked to long-term exposure to pesticides without adequate protective measures. Our study also highlights the gap in safety knowledge and training among farmers, highlighting the importance of targeted educational interventions to mitigate health risks. However, some studies suggest that awareness of pesticide hazards does not always translate into safe practices. For instance, Pengpan found that even when farmers are aware of the risks associated with pesticide exposure, they often fail to implement protective measures effectively[36]. This complexity indicates that simply increasing awareness may not be sufficient; practical solutions and behavioral changes are necessary to improve health outcomes among farmers.

Safety Training and Policy

The findings regarding the ineffectiveness of training and education programs for farmers, particularly concerning the safe use of chemicals, highlight significant barriers to participation and engagement. Despite the efforts of extension officers and organizations to provide training, issues such as inconvenient schedules, perceptions of safety measures as tedious, and reluctance to attend safety-focused sessions persist. This situation calls for a reassessment of training approaches to better align with farmers' needs. The literature supports these findings,

indicating that training programs often fail to achieve their intended outcomes due to similar barriers. For instance, Rahaman et al. emphasize the importance of the motivation of farmers and extension staff in enhancing pesticide safety practices. They argue that without addressing the underlying barriers to participation, such as scheduling and perceived relevance, training programs will continue to fall short[37]. Similarly, Tandi et al. note that many farmers lack knowledge about appropriate pesticide management techniques, which can be exacerbated by poorly designed training programs that do not consider farmers' practical realities[38]. This suggests that a more tailored approach to education, considering the farmers' context and preferences, is essential for improving engagement and compliance with safety measures.

Moreover, the absence of safety policies and robust monitoring systems for the safe use of chemicals and farmer health is a critical issue that resonates with the literature. Nicolopoulou-Stamati et al. argue for the need for an agricultural concept that emphasizes reducing chemical pesticide use and implementing effective monitoring systems to safeguard human health and the environment[39]. This aligns with the findings that without clear guidelines and enforcement mechanisms, farmers are left to manage risks independently, often prioritizing productivity over safety. Furthermore, Wu et al. discuss the need for improved environmental policy regulation and enforcement to address the overuse of agricultural chemicals. They suggest that without effective policies and monitoring, the risks associated with chemical use will continue to escalate[40]. This is echoed by Jepson et al., who highlight the widespread and severe risks associated with pesticide use, emphasizing the need for an enabling environment that supports sustainable agricultural practices[41]. Our findings underscore this gap, indicating a pressing need for comprehensive policies that not only promote safe chemical use but also ensure that farmers have the necessary support and resources to comply with safety standards. In contrast, while the literature emphasizes the importance of policy and monitoring, some studies suggest that simply implementing policies is insufficient without addressing the

underlying cultural and practical challenges faced by farmers. For example, Khakhula points out that a lack of stakeholder confidence and distorted institutional frameworks can hinder the effective implementation of agricultural policies[42]. This indicates that while robust policies are necessary, they must be accompanied by efforts to build trust and capacity among farmers and stakeholders to be effective.

Food Safety and Use of Toxic Chemicals

The findings regarding the lack of quality control in locally sold produce and the resulting food safety concerns highlight a significant issue in consumer confidence. Participants expressed a preference for imported goods, believing them to be safer than local alternatives, which underscores the need for enhanced quality assurance measures for local crops. This situation is consistent with existing literature that addresses consumer perceptions of food safety and the implications for local agricultural practices. Research by Scarpato et al. emphasizes that consumers are increasingly concerned about food safety, which significantly influences their purchasing decisions[43]. They argue that policy strategies are essential to reassure consumers about the safety of food products. This aligns with our findings, where the lack of routine testing for chemical residues in locally grown crops has led to diminished consumer trust. The perception that imported goods are safer reflects a broader trend where consumers prioritize safety over local sourcing, as noted by Obayelu et al., who found that health risks associated with toxic pesticide use in vegetable production contribute to consumer hesitance regarding local produce[44]. Moreover, the literature indicates that the absence of quality control measures can lead to significant public health risks. For instance, Xi et al. discuss the increasing concern over food safety incidents and the need for regulatory authorities to implement stringent quality and safety supervision in agricultural production. This finding resonates with the current study's emphasis on the necessity for robust quality assurance measures to ensure that both local and export crops meet safety standards[45].

Recommendations and Limitations

The research underscores the need for concerted efforts to foster collaboration, advocate for policy reforms, and mobilize resources to support the sustainable development of agriculture in Trinidad and Tobago. Recommendations involve education and adherence to safety protocols to address key issues related to occupational safety and health in the sector. While the implementation of guidelines for occupational safety and health may offer some benefits, its effectiveness hinges on various factors, including enforcement, awareness, and accountability. There should be collaboration between the ASTT and the MALF. Collaboration is critical for addressing the myriad challenges faced by farmers and for providing effective support and resources. A vision of partnership and cooperation between government entities and the agriculture community, highlighting the potential for collective action to drive positive change and enhance the resilience of the sector.

One limitation of our study is that it relied on self-reported data, which may be subject to recall bias or selective reporting. Participants may not have fully disclosed unsafe practices or health concerns due to social desirability bias, leading to potential underreporting of issues related to chemical exposure. Additionally, our study focused on a specific group of farmers and Ministry of Agriculture representatives in Trinidad and Tobago, which may limit the generalizability of the findings to other regions or contexts with different agricultural practices and regulatory environments. The absence of triangulation with quantitative data or direct observation of farming practices further constrains the study's ability to verify participants' accounts.

CONCLUSIONS

In conclusion, the findings and literature collectively emphasize the urgent need for comprehensive strategies that integrate health and safety training, effective policy implementation, and consumer education to address the challenges faced in agricultural

practices. By prioritizing these areas, stakeholders can work towards creating a safer and more sustainable agricultural landscape that benefits farmers and consumers alike.

DATA AVAILABILITY

The datasets generated during and/or analyzed during the current study will be made available by the corresponding author upon acceptance.

CONFLICT OF INTEREST

The authors declare that they have no conflicts of interest in the research.

PROTECTION OF HUMAN AND ANIMAL SUBJECTS

No human subjects were involved in the project.

REFERENCES

1. Pesticides are “global human rights concern”, say UN experts urging new treaty. *OHCHR* https://www.ohchr.org/en/press-releases/2017/03/pesticides-are-global-human-rights-concern-say-un-experts-urging-new-treaty.
2. Boedeker, W., Watts, M., Clausing, P. & Marquez, E. The global distribution of acute unintentional pesticide poisoning: estimations based on a systematic review. *BMC Public Health* **20**, 1875 (2020).
3. Nazarihaghighipashaki, M. & Moen, B. E. O-19 Fatal occupational injuries in farming, fishing and forestry occupations in southeast Asia, Australia, and selected European coastal countries, 2010–2015. *Occupational and Environmental Medicine* **80**, A17–A17 (2023).
4. Sunisa, C., Chuthamas, C. & Pornnapa, S. Situations of work-related diseases and injuries among agriculturists in the upper northeast regions of Thailand. *F1000Research* **11**, (2023).

5. Richardson, J. R., Fitsanakis, V., Westerink, R. H. S. & Kanthasamy, A. G. Neurotoxicity of pesticides. *Acta Neuropathol* **138**, 343–362 (2019).
6. Chen, W.-L. *et al.* Cancer risks in a population-based study of agricultural workers: results from the Taiwan's Farmers and Health Cohort study. *Scandinavian Journal of Work, Environment & Health* **49**, 419-427,419A (2023).
7. Benavente, Y. *et al.* Occupational exposure to pesticides and chronic lymphocytic leukaemia in the MCC-Spain study. *International Journal of Environmental Research and Public Health* **17**, (2020).
8. Elbaz, A. & Moisan, F. The scientific bases to consider Parkinson's disease an occupational disease in agriculture professionals exposed to pesticides in France. *Journal of Epidemiology and Community Health* **70**, 319 (2016).
9. Finhler, S. *et al.* Influence of pesticide exposure on farmers' cognition: A systematic review. *J Neurosci Rural Pract* **14**, 574–581 (2023).
10. van den Berg, H. *et al.* Pesticide lifecycle management in agriculture and public health: Where are the gaps? *Science of The Total Environment* **742**, 140598 (2020).
11. Kubiak-Hardiman, P. *et al.* Identifying Gaps and Challenges in Global Pesticide Legislation that Impact the Protection of Consumer Health: Rice as a Case Study. *Exposure and Health* **15**, 597–618 (2023).
12. Maraj, S. & Ganpat, W. Prevalence of acute pesticide poisoning in Trinidad. *Tropical Agriculture* (2018).
13. Pesticide use in agriculture a concern for Gov't | Loop Trinidad & Tobago. *Loop News* https://tt.loopnews.com/content/pesticide-use-agriculture-concern-govt.
14. Verteuil, P. D. & Isaac, W.-A. P. Risk Factors for Chronic and Acute Pesticide Poisoning among Waged and Licensed Farm Workers in Rural Trinidad and Tobago. *Journal of Rural and Community Development* **11**, (2016).

15. World Bank Open Data. *World Bank Open Data* https://data.worldbank.org.
16. Our Ministry. *Ministry of Agriculture, Land and Fisheries* https://agriculture.gov.tt/about-us/.
17. Agricultural Society of Trinidad and Tobago. *Ministry of Agriculture, Land and Fisheries* https://agriculture.gov.tt/agencies-partners/agencies/agricultural-society-of-trinidad-and-tobago/.
18. Introducing Whisper. https://openai.com/index/whisper/.
19. Pohontsch, N. J. *et al.* Experiences of patients with advanced chronic diseases and their associates with a structured palliative care nurse visit followed by an interprofessional case conference in primary care – a deductive-inductive content analysis based on qualitative interviews (KOPAL-Study). *BMC Primary Care* **25**, 1–9 (2024).
20. Widianto, E. P., Suhari, S., Fibriansari, R. D. & Maisyaroh, A. ANALYSIS OF FARMERS' INTERNAL FACTORS WITH THE ABILITY TO KNOW HAZARDOUS MATERIALS. *Nurse and Health: Jurnal Keperawatan* **9**, 32–41 (2020).
21. Imoro, Z. A., Larbi, J. & Duwiejuah, A. B. Pesticide Availability and Usage by Farmers in the Northern Region of Ghana. *Journal of Health and Pollution* **9**, 190906 (2019).
22. Satya Sai, M. V. *et al.* Knowledge and Perception of Farmers Regarding Pesticide Usage in a Rural Farming Village, Southern India. *Indian J Occup Environ Med* **23**, 32–36 (2019).
23. Odisho, S. K. & Mohammad, F. K. Blood cholinesterase activities and oxidative stress status among farmworkers using pesticides in Duhok, KRG, Iraq. *Journal of Ideas in Health* **5**, (2022).
24. Rosanti, E., Rahma, R. A. A., Hamawi, M. & Arifah, D. A. Farmers Knowledge on Pesticide Safety Management in Ponorogo. in 17–20 (Atlantis Press, 2021). doi:10.2991/ahsr.k.210115.004.

25. Damalas, C. A. & Koutroubas, S. D. Farmers' Training on Pesticide Use Is Associated with Elevated Safety Behavior. *Toxics* **5**, 19 (2017).

26. The Relationship of Knowledge and the Use Of Personal Protective Equipment Among Farmer Users of Pesticides in Waru Village, Slogohimo District | Wisnu | Contagion: Scientific Periodical Journal of Public Health and Coastal Health. https://jurnal.uinsu.ac.id/index.php/contagion/article/view/17694.

27. Oyekale, A. S. Cocoa Farmers' Compliance with Safety Precautions in Spraying Agrochemicals and Use of Personal Protective Equipment (PPE) in Cameroon. *International Journal of Environmental Research and Public Health* **15**, 327 (2018).

28. Mergia, M., Weldemariam, E. D., Eklo, O. M. & Yimer, G. T. Knowledge, Attitude, and Practice of Farmers on Pesticide Use and Their Impacts on the Environment and Human Health From Small Scale Vegetable Farming Along the Littoral of Lake Ziway, Ethiopia. Preprint at https://doi.org/10.21203/rs.3.rs-139366/v1 (2021).

29. Hashemi, S. M., Hosseini, S. M. & Hashemi, M. K. Farmers' perceptions of safe use of pesticides: determinants and training needs. *Int Arch Occup Environ Health* **85**, 57–66 (2012).

30. Mohamed, A. O. *et al.* KNOWLEDGE, ATTITUDES AND PRACTICES OF FARMERS TOWARDS PESTICIDES USE AND HANDLING IN GREENHOUSE FARMS, SUDAN. *International Journal of Research -GRANTHAALAYAH* **6**, 520–534 (2018).

31. Negatu, B., Dugassa, S. & Mekonnen, Y. Environmental and Health Risks of Pesticide Use in Ethiopia. *Journal of Health and Pollution* **11**, 210601 (2021).

32. Pesticide Knowledge and Safety Practices among Farm Workers in Kuwait: Results of a Survey - PubMed. https://pubmed.ncbi.nlm.nih.gov/28338612/.

33. Kapeleka, J. A., Sauli, E., Sadik, O. & Ndakidemi, P. A. Biomonitoring of Acetylcholinesterase (AChE) Activity among Smallholder Horticultural Farmers

Occupationally Exposed to Mixtures of Pesticides in Tanzania. *Journal of Environmental and Public Health* **2019**, 3084501 (2019).

34. Peasant association member's knowledge, attitudes, and practices towards safe use of pesticide management - Karunamoorthi - 2011 - American Journal of Industrial Medicine - Wiley Online Library. https://onlinelibrary.wiley.com/doi/10.1002/ajim.21008.
35. Fibriansari, R. D., Maisyaroh, A. & Widianto, E. P. The Nursing Assessment of Work History in Agricultural Areas with Liver and Kidney Functions in Farmers. *NurseLine Journal* **8**, 1–8 (2023).
36. Pengpan, R. *et al.* Relationship Between Pesticide Exposure Factors and Health Symptoms Among Chili Farmers in Northeast Thailand. *JPMPH* **57**, 73–82 (2023).
37. Rahaman, M. M., Islam, K. S. & Jahan, M. Rice Farmers' Knowledge of the Risks of Pesticide Use in Bangladesh. *Journal of Health and Pollution* **8**, 181203 (2018).
38. Small-Scale Tomato Cultivators' Perception on Pesticides Usage and Practices in Buea Cameroon. https://www.scirp.org/journal/paperinformation?paperid=52552.
39. Nicolopoulou-Stamati, P., Maipas, S., Kotampasi, C., Stamatis, P. & Hens, L. Chemical Pesticides and Human Health: The Urgent Need for a New Concept in Agriculture. *Front. Public Health* **4**, (2016).
40. Wu, Y. *et al.* Policy distortions, farm size, and the overuse of agricultural chemicals in China. *Proceedings of the National Academy of Sciences* **115**, 7010–7015 (2018).
41. Jepson, P. C. *et al.* Measuring pesticide ecological and health risks in West African agriculture to establish an enabling environment for sustainable intensification. *Philosophical Transactions of the Royal Society B: Biological Sciences* **369**, 20130491 (2014).

42. Khakhula, B., Kostyuk, O., Lanchenko, O., Antonyuk, H. & Homon, O. Innovative tools for risk management of the production activities of agricultural enterprises in an institutional environment. *SH* **2**, 136–153 (2024).

43. Scarpato, D., Rotondo, G., Simeone, M., Gómez, A. & Gutiérrez, P. How can food companies attract the consumer concerned about food safety? A logit model analysis in Spain. *British Food Journal* **119**, 1705–1717 (2017).

44. Consumers' Perception and Willingness to Pay for Organic Leafy Vegetables in Urban Oyo State, Nigeria. https://journalejnfs.com/index.php/EJNFS/article/view/229.

45. Xi, W. D., Qiao, Q. & Sun, S. F. The Optimization and Countermeasures Research of Agricultural Products Quality and Safety Supervision Mode. *Advanced Materials Research* **1061–1062**, 1271–1274 (2015).
