# Supplementary material for: Occupational health risk of farmers: A qualitative study with the agriculture society of trinidad and tobago and the ministry of agriculture, land and fisheries
Source: PLoS One. 2025 Jul 2;20(7):e0327283. doi: 10.1371/journal.pone.0327283 (PMC12221002; doi:10.1371/journal.pone.0327283)
Supplement: Attachment — Submitted filename: Avishek Choudhury et al.docx [file pone.0327283.s002.docx]

The manuscript titled “**Occupational Health Risk of Farmers: A Qualitative Study with the Agriculture Society of Trinidad and Tobago and the Ministry of Agriculture, Land and Fisheries.**”

After carefully reviewing the manuscript, I find the methodology to be sound and the writing well-structured. Therefore, I recommend minor revisions before acceptance. Below are my comments.

**Strength of the manuscript**

The manuscript addresses a crucial public health issue by examining occupational health risks among farmers due to pesticide exposure. Its focus on Trinidad and Tobago provides valuable regional insight. By incorporating interviews with participants from various stakeholder groups involved in pesticide use, the study offers a comprehensive and in-depth analysis.

**Things to improve**

1. Line 107 and 108 – “Its 10.5% of the land area is designated as agricultural land with a contribution of 1.07% towards GDP.” This sentence need clarity.
2. Line 123 – which problem was identified and which area of research?
3. Did the participants provide signed consent?
4. How was inductive and deductive phase of coding done?
5. How can the result in this study serve as a basis for further quantitative research?
